# Angiotensin‐converting enzyme 2 modulation of pyroptosis pathway in traumatic brain injury: A potential therapeutic target

**DOI:** 10.1002/ctm2.70167

**Published:** 2024-12-31

**Authors:** Jinxiu Guo, Shiyuan Zhao, Xue Chu, Changshui Wang, Junjun Meng, Shanshan Wei, Jianhua Wang, Yujin Guo, Weihua Kong, Wenxue Sun, Tao Zhang, Ruili Dang, Mengqi Yang, Jing Chen, Pei Jiang

**Affiliations:** ^1^ Institute of Clinical Pharmacy Jining First People's Hospital Shandong First Medical University Jining P.R. China; ^2^ Translational Pharmaceutical Laboratory Jining First People's Hospital Shandong First Medical University Jining P.R. China; ^3^ Department of Neurosurgery Affiliated Hospital of Jining Medical University Jining Medical University Jining P.R. China; ^4^ Department of Graduate Shandong Academy of Medical Sciences Shandong First Medical University Jinan P.R. China; ^5^ Institute of Central Nervous Vascular Injury and Repair Jining Academy of Medical Sciences Jining P.R. China; ^6^ Department of Pharmacy Shandong Provincial Hospital Affiliated to Shandong First Medical University Jinan P.R. China; ^7^ Neurobiology Key Laboratory of Jining Medical University Jining P.R. China; ^8^ Division of Biomedical Sciences Warwick Medical School University of Warwick Coventry UK

1

Dear Editor,

Traumatic brain injury (TBI) is a significant public health concern, with its severity largely influenced by secondary molecular damage like oxidative stress, cell death and neuroinflammation. ACE2 mediates the enzymatic conversion of AngII to Ang‐(1−7) and interacts with the G protein‐coupled receptor MasR, resulting in antagonistic biological effects to those of AngII. Research has shown that the ACE2/Ang‐(1–7)/MasR pathway mitigates neuroimmune overactivation, thereby decreasing neural damage and brain inflammation associated with cerebral haemorrhage and ischemia‐reperfusion.^1^ Understanding the regulation of ACE2 could provide novel insights into its neuroprotective mechanisms of ACE2 and offer fundamental knowledge regarding its underlying molecular signalling pathways. Following TBI, ACE2 levels in the injured cortical area significantly decreased (Figure 1A), reaching their lowest point 24 h post‐injury (Figure S1A,D). The ACE2 protein was found in MAP2, IBA1 and GFAP‐positive brain cells (Figure 1B,C), with co‐expression analysis of ACE2 and IBA1 further supporting this conclusion (Figure 1H–J). Additionally, TBI disrupts the normal function of the renin‐angiotensin system (Figure 1D–G). Behavioural experiments were verified to confirm the neuroprotective effect of ACE2 in vivo (Figure 1K). AVE0991, a synthetic Mas receptor agonist, replicates Angiotensin‐(1−7) effects by activating MasR and providing anti‐inflammatory, anti‐oxidative and anti‐apoptotic benefits. AVE0991 treatments significantly mitigated cognitive decline caused by TBI, demonstrated by decreased escape latency time and distance in the learning curve (Figure 1L–N). Additionally, AVE0991 improved motor coordination and balance in TBI mice, evidenced by shorter completion times in the horizontal ladder and balance beam tests and better scores (Figure 1O–R). CRISPR/Cas9 successfully constructed a mouse ACE2‐knockout (KO) model (Figure S1B–F). ACE2‐KO exacerbated behavioural impairment in mice following TBI (Figure S1H–N).

**FIGURE 1:**
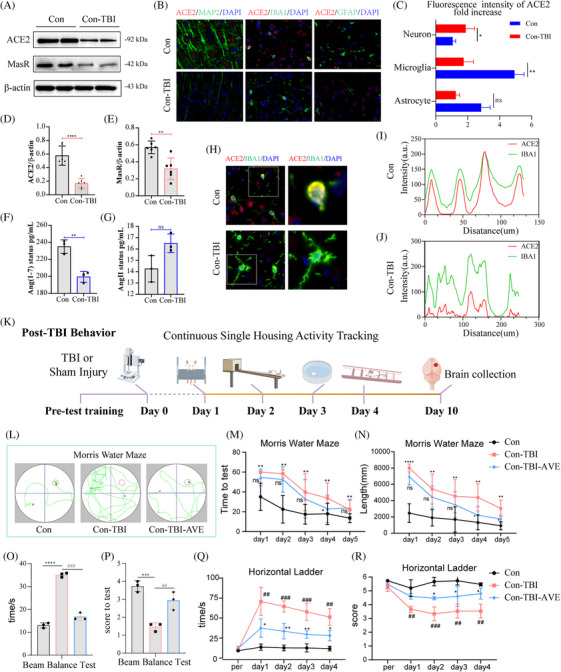
TBI‐induced renin‐angiotensin system impairment affects exercise capacity and cognitive function in mice. (A) ACE2 expression levels 24 h post‐brain injury. (B) Immunofluorescence staining to determine the cellular localization of ACE2 protein within the brain. (C) Fluorescence intensity analysis revealed the most significant differential ACE2 expression in microglia following brain injury. (D–G) Immunoblotting and ELISA validation demonstrated dysregulation of the renin‐angiotensin system (RAS) post‐brain injury. (H–J) Co‐expression analysis of ACE2 and IBA1 highlighted the effects of TBI on ACE2 expression in microglia. (K) Schematic representation of behavioural experiments conducted on mice post‐TBI. (L) Representative traces indicate the mice's paths during the spatial exploration and directional navigation tests. (M) AVE0991 treatment reduced the time required to locate the submerged platform and (N) shortened the distance covered in the platform quadrant exploration. (O–R) AVE0991 improved motor coordination and balance in mice with brain injury, as evidenced by shorter completion times in horizontal ladder and balance beam tests and enhanced motor scores.

Pyroptosis, a recently identified form of inflammatory cell death, has been linked to various central nervous system disorders, including TBI.^2^, ^3^ To investigate the impact of ACE2 depletion on TBI progression, transcriptome sequencing was performed, revealing 385 upregulated and 74 downregulated genes (Figure 2A). Enrichment analysis of these differentially expressed genes using Reactome highlighted the pyroptosis pathway (Figure 2B), with significant changes observed in pyroptosis‐related factors such as Gsdmd, Casp1, Il18rap and illr2. The quantitative reverse transcriptase PCR (qRT‐PCR) validation confirmed the upregulation of messenger ribonucleic acid levels for this pyroptosis (Figure S2C). Protein imprinting assessments revealed that ACE2 deletion exacerbated the activation of key pyroptosis factors GSDMD and CASP1 (Figure 2C–E), findings further supported by immunofluorescence double staining (Figure 2I). The absence of ACE2 significantly increased the activation levels of mature pro‐inflammatory cytokines IL‐1β and IL‐18, both implicated in pyroptosis (Figure S2D–I). Sholl analysis, used to assess morphological changes in pericontusional microglial cells post‐TBI, demonstrated a transition from a resting to an activated state (Figure 2F–H).

**FIGURE 2:**
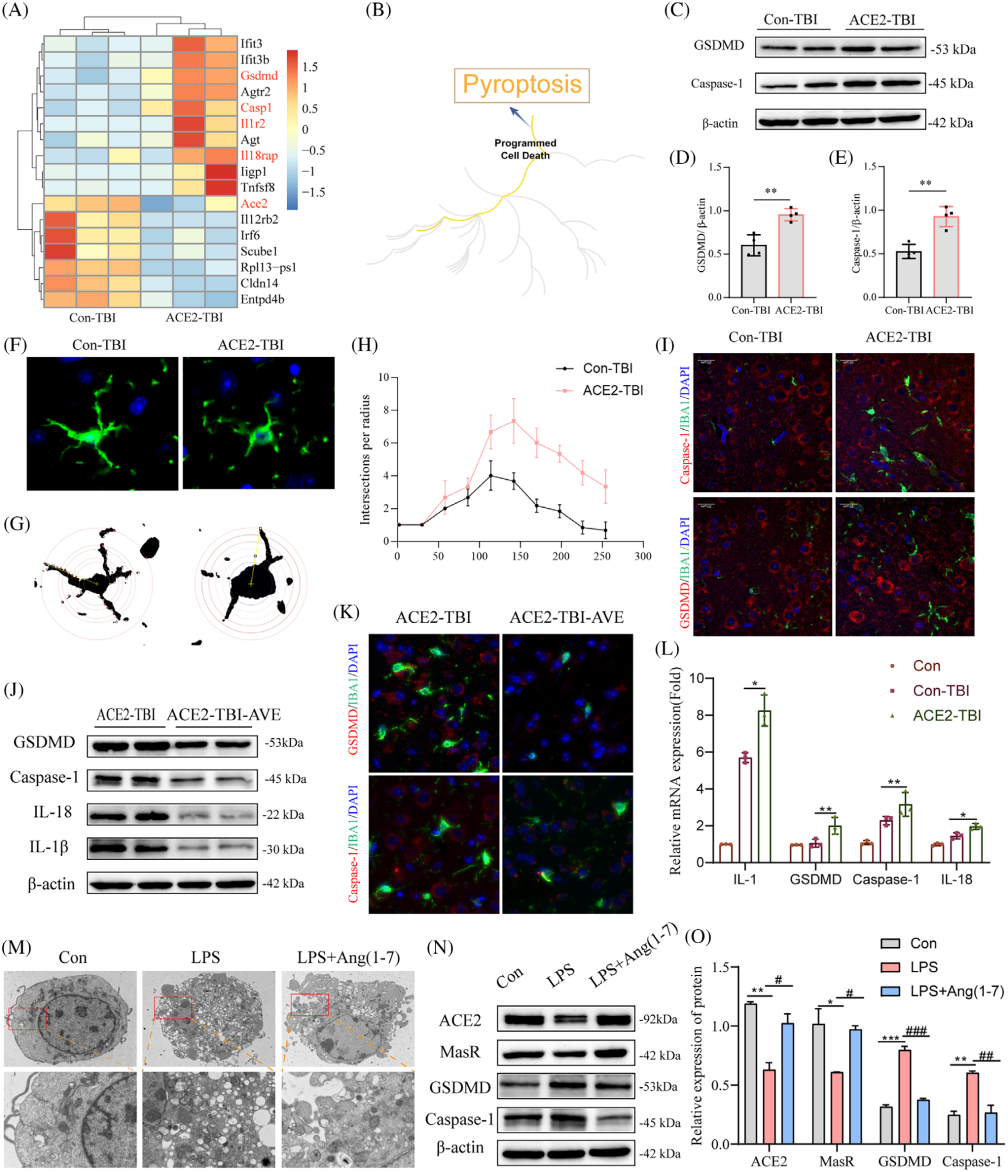
Impact of ACE2 depletion on cell pyroptosis in TBI and intervention effects of ACE2 agonist AVE0991 and Ang‐(1–7). (A) Heatmap visualization of differentially expressed gene expression levels. (B) Enrichment analysis of differentially expressed genes in Reactome highlighting the pyroptosis pathway. (C–E) Western blot validation of the activation of pyroptosis markers GSDMD and Caspase‐1, with findings indicating that ACE2 deficiency exacerbates cell pyroptosis. (F–H) Sholl analysis evaluating microglial activation post‐brain injury. (I) Co‐localization of microglial marker IBA1 with pyroptosis factors. (J) Western blot validation demonstrates that AVE0991 mitigates ACE2 deficiency‐induced cell pyroptosis and inflammation. (K) AVE0991 reduces the fluorescence intensity of pyroptosis factors. (L) Validation of pyroptosis and inflammation‐related genes through qRT‐PCR. (M) Transmission electron microscopy demonstrates that Ang‐(1–7) significantly alleviates LPS‐induced cell pyroptosis. (N) Western blot analysis and (O) corresponding analysis further confirm this conclusion. Data are represented as the mean ± scanning electron microscope (SEM) of three independent experiments.

To further elucidate the role of ACE2 in TBI‐induced pyroptosis,^4^, ^5^, ^6^ we administered the ACE2 agonist AVE0991 to mice. AVE0991 treatment upregulated the expression levels of ACE2 and MasR, leading to an amelioration of cell pyroptosis (Figure S2J–N). Notably, AVE0991 demonstrated favourable outcomes in both animal models (Figure 2J–L). Lipopolysaccharide (LPS)‐induced pyroptosis models provide a controlled and reproducible system to study inflammasome activation and inflammatory responses central to TBI pathogenesis. Moreover, we utilized LPS to induce a cellular pyroptosis model and supplemented it with Ang‐(1–7) as an ACE2 adjunct. Transmission electron microscopy revealed cellular disintegration in the LPS group, characterized by extensive membrane rupture, cytoplasmic leakage, nuclear disintegration and chromatin condensation (Figure 2M). Ang‐(1−7) primarily exerts protective effects by binding to the MasR and activating downstream anti‐inflammatory signalling pathways. Conversely, Ang‐(1–7) significantly alleviated LPS‐induced cellular pyroptosis (Figure 2N). Assessment with nucleic acid dyes further corroborated the mitigating effect of Ang‐(1–7) on cell pyroptosis severity (Figure S2P).

To explore the connection between ACE2 downregulation or deficiency and cell pyroptosis, we employed omics approaches and bioinformatics to uncover potential molecular mechanisms. Non‐targeted metabolomics analysis identified 134 distinct metabolites, as illustrated in the volcano plot (Figure S3A–C). Notably, the metabolite thromboxane B2 (TXB2) was significantly elevated in the ACE2‐TBI group, implicating the involvement of the acid metabolic pathway in pyroptosis regulation (Figure S3D). This pathway was further substantiated through lipid metabolism metabolomics analysis (Figure S4A,B), indicating a recurrence of differential lipid metabolites within the arachidonic acid metabolism pathway (Figure S3F). Notably, prostacyclin synthase (PTGIS) is a key metabolic enzyme in arachidonic acid metabolism,^7^, ^8^ displayed a significant downregulation in ACE2 deficiency (Figure 3A), corroborated by protein imprinting and immunohistochemistry (Figure S4E–G). Intriguingly, the Kyoto encyclopaedia of genes and genomes (KEGG) pathway analysis highlighted PTGIS as significantly enriched in the arachidonic acid metabolism pathway (Figure 3B). Collectively, these findings suggest that ACE2‐KO disrupts the expression of arachidonic acid metabolism products and enzymes, shedding light on the intricate interplay between ACE2 and cell pyroptosis regulation.

**FIGURE 3:**
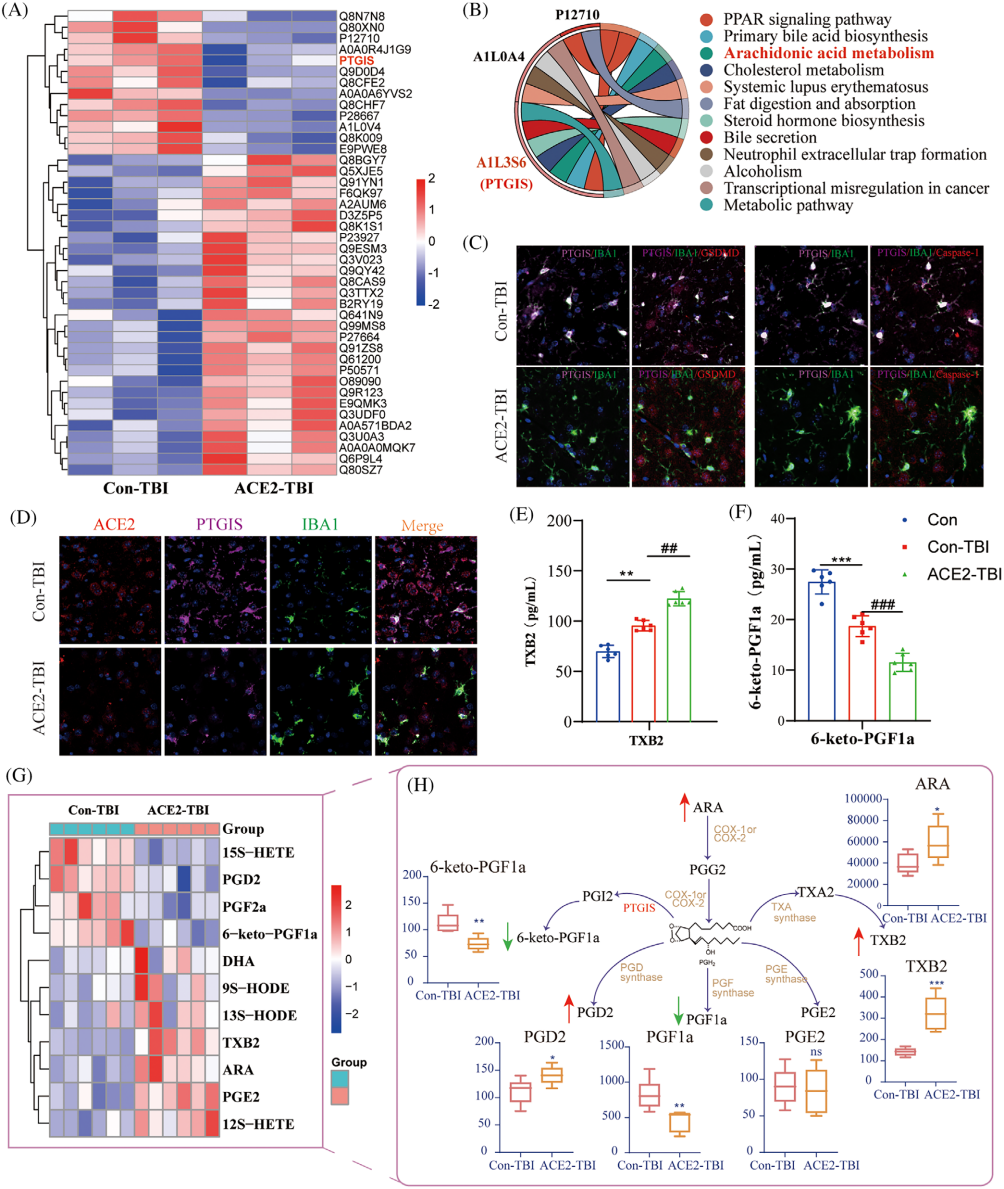
Impact of ACE2 deficiency on PTGIS expression and arachidonic acid metabolism in TBI progression. (A) Heatmap visualization of differentially expressed protein levels, with colours representing expression levels, ranging from red (high expression) to blue (low expression). (B) KEGG enrichment chord diagram, illustrating the distribution of proteins across KEGG pathways. (C) Immunofluorescence co‐expression analysis of PTGIS and pyroptosis markers in microglia. (D) Immunofluorescence co‐localization of ACE2 and PTGIS in microglia. (E–F) ELISA validation of dysregulated levels of arachidonic acid metabolites TXB2 and 6‐keto‐PGF1a. (G) Clustering heatmap of arachidonic acid‐targeted metabolomics results and quantitative result analysis (H). Red arrows indicate increased metabolic levels, while green arrows represent decreased metabolic levels. Data are presented as mean ± SEM from three independent experiments.

Our study explored the role of PTGIS in brain injury and its co‐expression with IBA1. The results indicated that PTGIS expression decreases as microglial activation increases following TBI (Figure 3D). Moreover, the ACE2‐TBI group exhibited a heightened level of pyroptosis alongside reduced PTGIS expression levels (Figure 3C). Enzyme‐linked immunosorbent assay (ELISA) results confirmed a significant increase in the arachidonic acid metabolite TXB2 following TBI in comparison to normal mice, with ACE2‐TBI mice exhibiting strong expression (Figure 3E). Conversely, low PTGIS expression led to a notable decrease in the levels of the PGI2 metabolite 6‐keto‐PGF1a (Figure 3F). The findings from arachidonic acid‐targeted metabolomics further supported our initial hypothesis, as illustrated by metabolic clustering diagrams and analysis (Figure 3G). As indicated in the diagram, red arrows denote elevations in metabolic levels, while green arrows represent reductions. The absence of ACE2 significantly disrupts the balance equilibrium between TXA2 and PGI2, exacerbating the pyroptosis mechanism (Figure 3H).

Based on the findings presented, it is hypothesized that supplementing with PTGIS may mitigate the dysregulated metabolism of arachidonic acid and reduce pyroptosis progression. LPS was used to induce cell pyroptosis, and a microglial PTGIS overexpression vector was utilized to assess the impact of PTGIS on pyroptosis (Figure 4A). PTGIS effectively suppressed the release of inflammatory mediators associated with pyroptosis, as well as the maturation of GSDMD and Caspase‐1 (Figure 4B). Scanning electron microscopy further confirmed that PTGIS inhibited pyroptosis progression and delayed cell disintegration (Figure 4C). This conclusion was further supported by immunofluorescence and transcript‐level analyses (Figure 4D,E). Although the inhibitory effect of LPS on 6‐keto‐PGF1a levels was not pronounced, PTGIS intervention restored these levels to normal (Figure 4F). PTGIS intervention promoted arachidonic acid metabolism, reduced cellular expression and normalized PGE2 levels. Nucleic acid dyes were utilized to assess cellular morphology. The findings indicated that LPS increased the permeability of the cellular membrane, resulting in the entry of YO‐PRO‐1 into the cell nucleus and an increase in fluorescence (Figure 4G). EthD‐2 was observed to penetrate the damaged membrane and bind to a greater amount of nucleic acids (Figure 4G). Besides, PTGIS was found to inhibit the process of pyroptosis.

**FIGURE 4:**
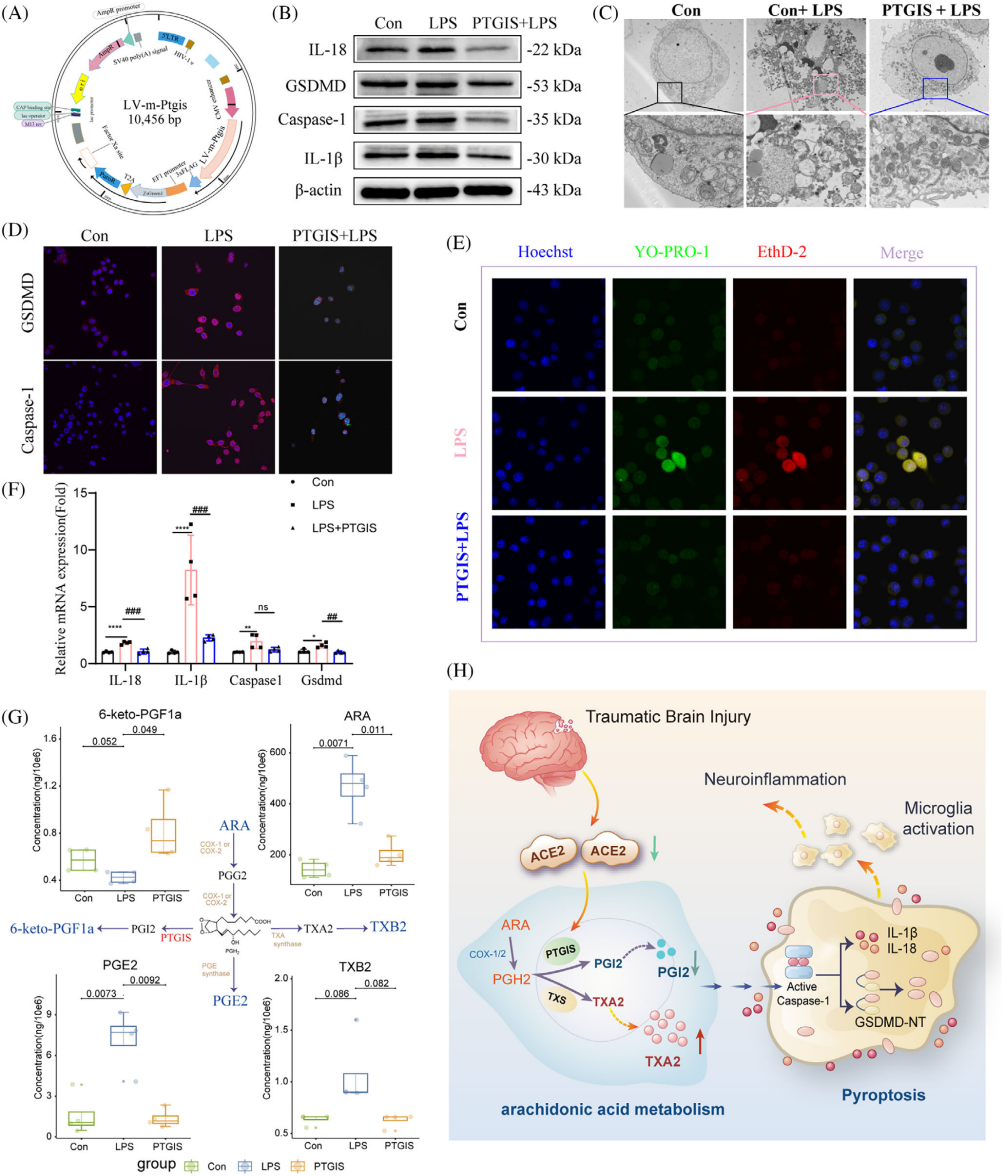
Investigation of the impact of PTGIS supplementation on cell pyroptosis and arachidonic acid metabolism. (A) Schematic representation of the construction of the PTGIS lentiviral overexpression vector. (B) Western blot validating the effect of PTGIS overexpression on LPS‐induced cell pyroptosis, along with (C) scanning electron microscopy highlighting the morphological impact of PTGIS on cell pyroptosis. (D) Immunofluorescence staining to detect GSDMD and CASPASE‐1 in PTGIS‐transfected microglia and (E) validation of gene expression for pyroptosis markers and inflammatory cytokines. (F) Arachidonic acid‐targeted metabolomics analysis confirming dysregulation in arachidonic acid metabolism at the cellular level. (G) Assessment of membrane damage in cell morphology using nucleic acid dyes. (H) A schematic diagram summarizing our findings on the mechanism. Data are represented as mean ± SEM of three independent experiments.

TXA2 serves as a counterbalance to PGI2. U46619, a TXA2 agonist, directly exacerbates cell pyroptosis,^9^ leading to increased cell membrane permeability and upregulating the expression of a pyroptosis‐related protein (Figure S5A,B,E,F). This progression is linked to dysregulation in the levels of TXB2 and 6‐keto‐PGF1a (Figure S5C−E). Ozagrel, a TXA2 inhibitor, was used to explore the role of TXA2 in TBI. In vivo, ozagrel administration to TBI mice significantly mitigated cognitive and motor deficits associated with TBI (Figure S5H–O). These results indicated that TXA2 signalling contributes to inflammation and pyroptosis following TBI, and inhibiting this pathway can help reduce these effects.

This study identified ACE2 deficiency as the initiator of disrupted TXA2/PTGIS balance, contributing to pyroptosis and inflammation (Figure 4H). Our research elucidates the dual function of ACE2 in attenuating inflammation and modulating pyroptosis through the metabolism of arachidonic acid, thereby expanding its recognized anti‐inflammatory properties. In contrast to the AngII/AT1R axis, which intensifies injury, the ACE2 axis alleviates inflammation and metabolic disturbances, directly impacting secondary injury mechanisms in TBI. Our findings distinctly associate the ACE2‐mediated restoration of PTGIS and the regulation of TXA2 levels with a reduction in pyroptosis, offering a novel perspective on its neuroprotective effects in TBI.

## AUTHOR CONTRIBUTIONS

Jinxiu Guo, Shiyuan Zhao, Jing Chen and Pei Jiang designed this experiment. Xue Chu, Shanshan Wei and Junjun Meng carried out animal models and performed the behaviour tests. Ruili Dang, Changshui Wang, Jinxiu Guo and Mengqi Yang conducted the biochemical experiments. Jinxiu Guo wrote this manuscript. Jianhua Wang, Yujin Guo, Weihua Kong, Mengqi Yang, Tao Zhang and Wenxue Sun carried out analysis and interpretation of the data. All authors have read and approved the final version of the manuscript.

## CONFLICT OF INTEREST STATEMENT

The authors declare no conflict of interest.

## FUNDING INFORMATION

The study was supported by the National Natural Science Foundation of China (No. 82272253), the Natural Science Foundation of Shandong Province (No. ZR2022MH007) and the Key R&D Program of Jining (No. 2023YXNS016, No. 2022YXNS148 and No. 2023YXNS037).

## ETHICAL APPROVAL STATEMENT

All animal studies were conducted following the Care and Use of Laboratory Animals, with the approval of the Ethics Committee of Jining First People's Hospital (JNRM‐2022‐DW‐011).

## Supporting information



Supporting Information

## Data Availability

All data supporting the findings of this study are available from the corresponding author upon reasonable request.
