# Supplementary material for: Angiotensin‐converting enzyme 2 modulation of pyroptosis pathway in traumatic brain injury: A potential therapeutic target
Source: Clin Transl Med. 2024 Dec 31;15(1):e70167. doi: 10.1002/ctm2.70167 (PMC11686427; doi:10.1002/ctm2.70167)
Supplement: Supplementary file 1 — Supporting Information [file CTM2-15-e70167-s001.docx]

**ACE2 Modulation of Pyroptosis Pathway in Traumatic Brain Injury: A Potential Therapeutic Target**

**Material and Method**

**1 Primary cell cultures**

Newborn C57BL/6J mice aged 24 hours were used for the experiment. The bilateral cerebral cortices were isolated and mechanically sheared under sterile conditions. The cells were then separated using a combination of mechanical shearing and layered pipetting, followed by filtration. After centrifugation, cells were resuspended in DMEM/F12 medium supplemented with 10% fetal bovine serum and seeded into poly-D-lysine-coated culture flasks. The flasks were then placed in a 37°C incubator with 5% CO2. The mixed glial cells were cultured for approximately 7 days until they reached optimal growth[20].

**2 Animal**

Sex was not considered as a biological variable. Our study examined male mice. Male C57BL/6J mice weighing 20-25 g and aged 6-8 weeks were obtained from Jinan PengYue Laboratory Animal Breeding Co., Ltd (Jinan, China). ACE2-KO mice on a C57BL/6J background were bred by SPF (Beijing, China) Biotechnology Co., Ltd. The mice were housed in a 12-hour light/dark cycle with a constant temperature of 23°C ± 2°C, and provided ad libitum access to food and water. Prior to the experiment, the mice were acclimated to their environment for 7 days. The mice were randomly assigned to either the operation (TBI) group or the sham-operation (Con) group or drug treatment (AVE) group.

**3 TBI model**

The TBI model used in our study is the controlled cortical impact (CCI) model, a well-established approach for inducing traumatic brain injury in mice. This method enables consistent and reproducible injuries, simulating clinical TBI scenarios. Mice were anesthetized via intraperitoneal injection of 100 mg/kg sodium pentobarbital and maintained at a temperature of 37±0.5°C using a heating blanket. Subsequently, the mice were secured in a stereotaxic frame, and a 1.5-cm longitudinal midline scalp incision was made to expose the frontoparietal bones. A 3 mm diameter craniotomy was then performed on the right parietal bone, followed by the application of a vertical impact using an impactor under the conditions of 6.0±0.2m/s velocity, 50ms dwell time, and 1.4mm depth. Post-injury, the skin incision was sutured, and the mice were returned to their cage upon recovery from anesthesia. The study includes four animal models: normal mice (Con), ACE2-KO mice, TBI models of both groups (Con-TBI and ACE2-TBI), and AVE0991-treated TBI models (Con-TBI-AVE and ACE2-TBI-AVE).

**4 Drug treatment**

AVE0991 (3mg/kg) was administered to mice via intraperitoneal injection for a continuous period of 2 weeks in order to activate the ACE2/Ang(1–7)/MasR axis[21]. To modulate TXA2 signaling, the selective TXA2 inhibitor ozagrel (MedChemExpress, HY-B0428) and the TXA2 activator U46619 (Glpbio, GC13205) were administered daily at doses of 5 mg/kg and 30 mg/kg. This regimen was continued once daily for 7 days prior to TBI until euthanasia or Morris water maze (MWM) testing finished. The mortality rate following the TBI operation was approximately 20%.

**5 Lentiviral transfection**

Microglia in a healthy state were inoculated into a 24-well plate at a concentration of 1×10^5^ cells per well. The plate was subsequently incubated at 37°C in a 5% CO2 environment for an overnight period. The virus was thawed gradually on ice following its removal from cold storage. The virus was introduced for infection at a multiplicity of infection (MOI) of 20, with a virus titer (TU/mL) of 1×10^8^. After 24 hours of infection, fresh virus was applied for secondary infection to increase virus infection efficiency. GFP expression efficiency was initially assessed through fluorescence microscopy 48 hours post-infection. The medium was replaced with fresh complete culture medium containing an optimal concentration of Puromycin, and stably transduced cell lines were selected for further experimentation.

**6 Western blotting**

Western blotting was conducted following the protocol outlined in a prior study. Western blot analysis was carried out according to the methodology outlined in a prior investigation. The primary antibodies utilized were ACE2 (Abcam, ab108252, 1:1000), MasR (Proteintech, 20080-1-AP, 1:2000), IL-1β (Abcam, ab9722, 1:1000), PTGIS (Affinity, DF4745, 1:1000), GSDMD (Santa Cruz Biotechnology, SC-393581, 1:1000), Caspase1 (Santa Cruz Biotechnology, SC-56036, 1:1000), IL-18 (Proteintech, 10663-1-AP, 1:2000), and β-actin (Proteintech, 20536-1-AP, 1:5000). Protein expression levels were normalized to β-actin. Post-exposure data were analyzed with ImageJ Software and GraphPad Prism 8.

**7 Enzyme-linked immunosorbent assay (ELISA)**

ELISA kits for Ang II (CSB-E04495m), Ang (1-7) (CSB-E13763m), TXB2 (CSB-E08048m), and 6-keto-PGF1a (CSB-E09422m) were procured from CUSABIO. Each molecule was quantified following the manufacturer's guidelines.

**8 Immunohistochemical and immunofluorescence staining**

Following anesthesia, the experimental mice underwent perfusion with PBS and subsequently with 4% paraformaldehyde (PFA). The brain was promptly dissected, fixed in 4% PFA overnight, and subjected to dehydration in a sucrose gradient solution. Subsequently, the brain was rapidly frozen and sectioned into 10 μm thick slices for subsequent immunofluorescence staining. Brain slices were treated with 0.25% TritonX-100 for 25 minutes, followed by sealing with 2% bovine serum albumin (BSA) for 90 minutes, and subsequently incubated with specific primary antibodies overnight at 4 °C. The study employed antibodies utilized in this study included ACE2 (Proteintech, ab108252, 1:100), IL-1β (Santa Cruz Biotechnology, SC-12742, 1:100), PTGIS (Affinity, DF4745, 1:100), GSDMD (Santa Cruz Biotechnology, SC-393581, 1:100), Caspase-1 (Santa Cruz Biotechnology, SC-56036, 1:100), IL-18 (Proteintech, 10663-1-AP, 1:2000), MAP2(Cell Signaling Technology, 8707S, 1:200), GFAP(Santa Cruz Biotechnology, SC-33673, 1:100), and Iba1 (Abcam, ab178846, 1:200). The following day, brain slices and secondary antibodies underwent incubation at room temperature in the absence of light for a duration of 1 hour. Subsequently, the nuclei of the cells were re-stained with DAPI (1RV 1000m Sigma) for a period of 15 minutes. The resulting image was captured using an Olympus confocal fluorescence microscope. All images were obtained utilizing identical microscope settings and processed with consistent parameters. Subsequently, the images from three randomly chosen microscopic visual fields were subjected to analysis.

**9 Polymerase Chain Reaction and Quantitative Real‑Time Polymerase Chain Reaction**

Total RNA was extracted from tissues and cells using Trizol reagent (Invitrogen, Shanghai, China) following the manufacturer's instructions. RNA concentration was determined with a NanoDrop 2000 spectrophotometer (Thermo Scientific).cDNA was synthesized from total RNA using SuperScript III Reverse Transcriptase (Invitrogen, Shanghai, China).Quantitative real-time PCR was conducted using a Bio-Rad CFX96 Touch Real-Time PCR detection system.β-actin expression levels served as the internal standard, with experiments conducted in triplicate and repeated three times. Primers were procured from Origene, and their sequences are detailed in Table S1.

**Table 1** Real-time PCR primer sequences

| Gene | Direction | Primer sequences |
| --- | --- | --- |
| IL-1β | Forward Primer | TAGAAGGAAGTCAGACACCCACAGG |
|  | Reverse Primer | CACAGAAGGAAGATGGCACGACAG |
| IL-18 | Forward Primer | GGGTTCTCTGTGGTTCCATGC |
|  | Reverse Primer | CCTGATGCTGGAGGTTGCAG |
| Caspase-1 | Forward Primer | ACAAGGCACGGGACCTATG |
|  | Reverse Primer | TCCCAGTCAGTCCTGGAAATG |
| Gsdmd | Forward Primer | CCAGCATGGAAGCCTTAGAG |
|  | Reverse Primer | CAGAGTCGAGCACCAGACAC |
| β-actin | Forward Primer | AGAGGGAAATCGTGCGTGAC |
|  | Reverse Primer | CAATAGTGATGACCTGGCCGT |

**10 TEM**

Cells obtained through centrifugation were first fixed in electron microscope fixative for a period of 2-4 hours and subsequently embedded in a 1% agarose solution. Following fixation with 1% osmic acid in a 0.1M phosphate buffer (pH 7.4) for 2 hours at room temperature in the absence of light, the cells underwent a series of dehydration, infiltration, and embedding steps using alcohol and acetone solutions. The embedding plate was then polymerized at a temperature of 60°C for a duration of 48 hours, after which ultra-thin sections measuring 60-80nm were produced utilizing an ultramicrotome. The copper mesh underwent an 8-minute treatment with 2% uranyl acetate, followed by three rinses in 70% alcohol, and was then stained with 2.6% lead citrate for 8 minutes in a CO2-free environment. The sections were air-dried overnight prior to transmission electron microscope analysis.

**11 Behavioral analysis**

On the second day post-TBI, the neurological deficits in each mouse were assessed using the balance beam experiment, morris water maze (MWM) test, rotarod test and foot fault test. A cohort of seven mice was allocated to each experimental group. Two independent investigators, unaware of the experimental conditions, conducted these assessments. We utilized behavioral experiments, including the MWM, balance beam test, and horizontal ladder test, to evaluate the functional consequences of ACE2 modulation on cognitive and motor deficits post-TBI. The MWM assessed spatial learning and memory, which are commonly impaired in TBI patients. The balance beam and horizontal ladder tests were selected to evaluate motor coordination and balance, which are reflective of sensorimotor integration and cerebellar function, often affected after TBI.

Balance beam experiment

Three days before the establishment of the traumatic brain injury model, mice were trained to walk on a balance beam. The day after the establishment of the traumatic brain injury model, the mice were placed on a balance beam and their gait pattern was scored on a scale of 0 to 6. The mice were positioned at the initial point of a horizontal wooden bar measuring 0.9 × 0.9 × 50 cm, elevated 40 cm above the ground, with a dark goal box placed at the end to entice the mice to traverse the bar towards the secure environment. The duration of time required for the mice to complete the crossing was recorded. Each mouse underwent three sessions per day, with a 15-20 minute break between sessions, for a period of five consecutive days.

Foot fault test

The Foot Fault Test (FFT) was utilized to evaluate the mice's paw placement ability on a grid. The mice underwent a 3-day training period prior to the operation, with each training session lasting 5 minutes at least twice daily. The average performance of each mouse at the conclusion of the third day of training was established as the baseline. On the third day post-TBI, the mice were positioned on a horizontal metal grid, and the total number of steps taken and instances of left forelimb slippage within a 2-minute timeframe were documented.

Morris water maze (MWM) test

This study evaluates the spatial learning and memory capabilities of mice through a series of training sessions following the induction of TBI. Mice undergo five days of training, consisting of four sessions per day, prior to the formal experiment. The duration of time taken by mice to locate and stand on a submerged platform after entering the water is measured. Upon locating the platform, mice are permitted to remain on it for 10 seconds. The trial assesses the escape latency and total swimming distance during the spatial navigation test.

Rotarod Test

The Rotarod test was utilized to assess the balance and sensorimotor coordination of the mice. The mice were required to navigate a five-channel rotating apparatus, starting at 4 rpm and accelerating to 40 rpm over a 300-second period. Prior to testing, the mice underwent a two-day training protocol with sessions spaced 30 minutes apart. During the test, the mice were placed on the rods sequentially, and the average fall latency from three trials was measured. Behavioral assessments were conducted by individuals blinded to the experimental groups.

All experimental groups underwent baseline behavioral assessments before injury induction to exclude pre-existing differences. No significant variations in baseline performance were observed between groups, confirming that post-TBI behavioral differences were attributable to the experimental treatments and not pre-existing conditions. Improvements in escape latency and time spent in the target quadrant in the MWM suggest that ACE2 activation (via AVE0991) alleviates cognitive deficits. Similarly, reduced foot-faults and shorter completion times in the motor coordination tests indicate enhanced recovery of motor function.

**12 Statistics**

Data were analyzed using SPSS 22.0 (IBM Corp., New York, NY, USA). Data represent as mean standard deviation (SD). Result expressed as mean ± standard deviation (SD). Statistical analysis was conducted using unpaired Student's t-test and one-way ANOVA. Data were performed using GraphPad Prism 8.0 (GraphPad Software, USA). Statistical significance was determined at a threshold of p<0.05.

**Supplementary Figure Legends**


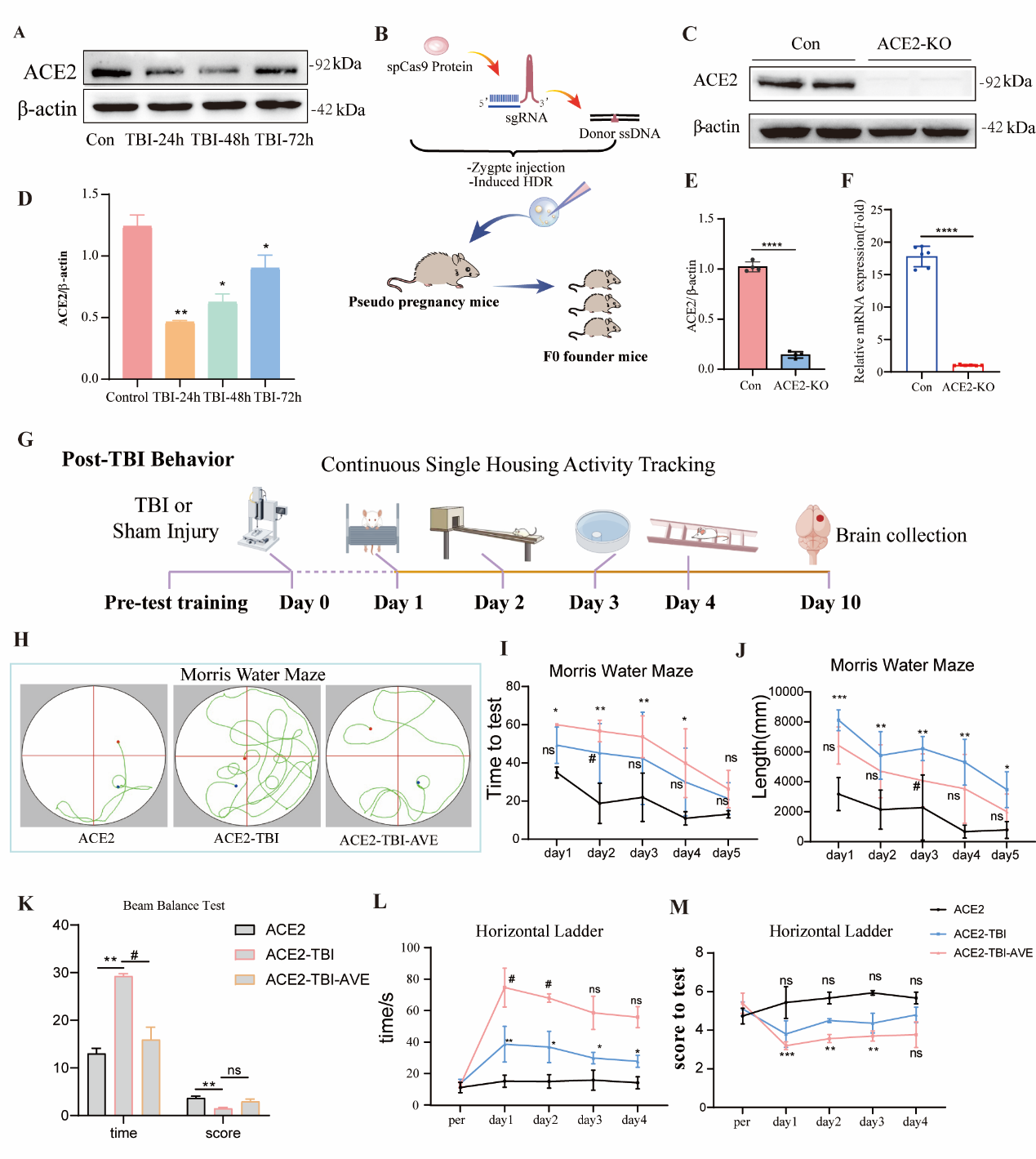


**Fig. S1. Influence of TBI on ACE2 Expression and its Improvement on Behavioral Performance in TBI Mice.**

(A and D) ACE2 expression levels reach their lowest point 24 h post-TBI. (B) Schematic representation of the construction of ACE2 knockout mice using CRISPR/CAS9 technology. (C, E, and F) Immunoblotting and PCR analysis confirm the successful knockout of ACE2 in mice. (G) Schematic illustration of behavioral experiments conducted post-TBI. (H) Representative traces indicate the paths of mice during spatial exploration and directional navigation tests. (I-J) AVE0991 treatment significantly reduces the time spent searching for the submerged platform and shortens the distance traveled within the platform quadrant. (K–M) AVE0991 improves motor coordination and balance in TBI mice, as evidenced by shorter completion times in the horizontal ladder and balance beam tests and improved motor scores.

**
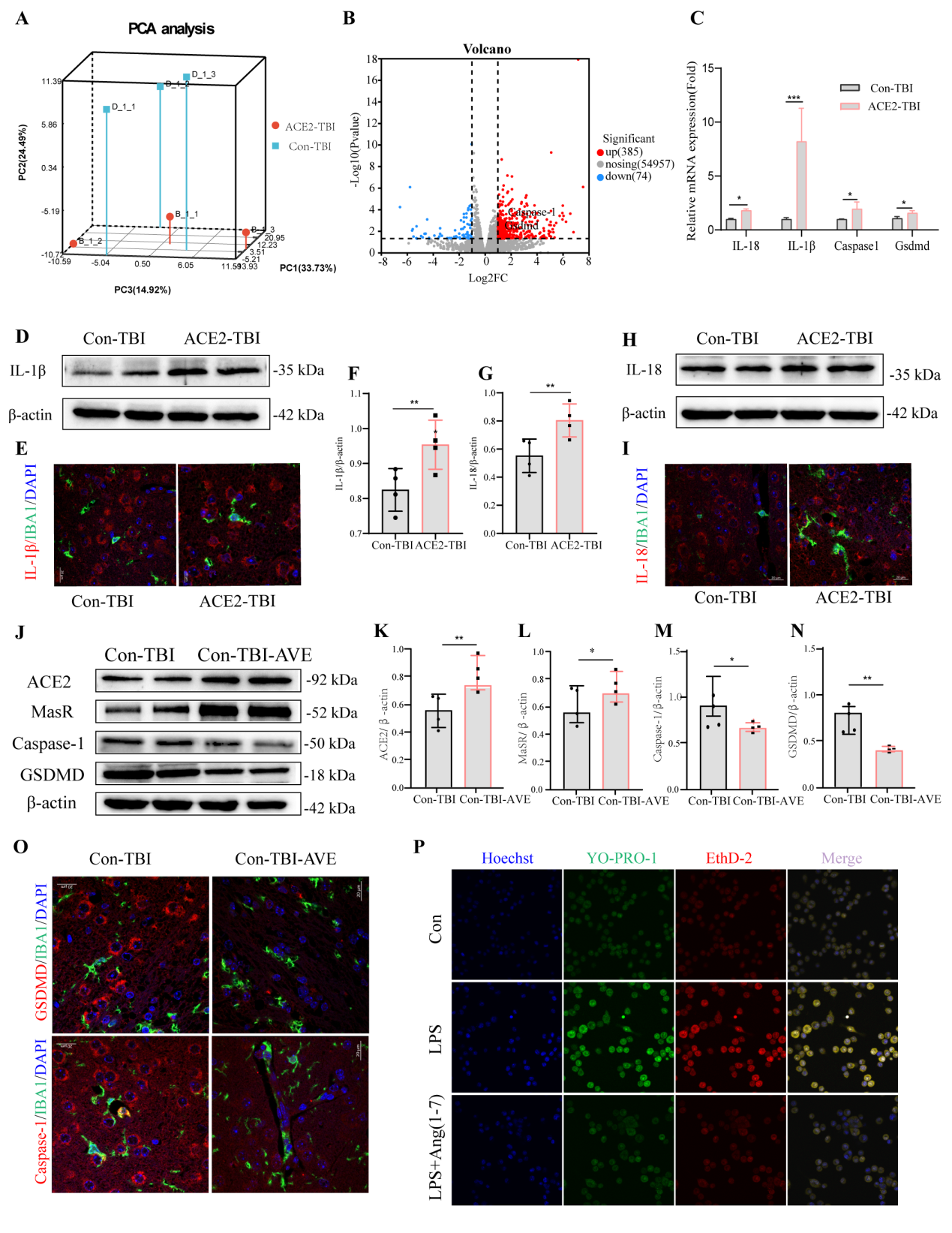
**

**Fig. S2**. **Impact of ACE2 Deletion on Post-TBI Inflammatory Factor Expression and AVE0991-Mediated Amelioration of Pyroptosis and Inflammation in TBI Mice.**

(A) Principal component analysis (PCA) of transcriptome data. (B) Volcano plot illustrating 385 upregulated and 74 downregulated genes. (C) PCR validation of the expression levels of inflammatory and pyroptosis-related factors. (D–I) Western blot analysis indicating elevated IL-1β and IL-18 levels in ACE2 knockout TBI mice. (J–N) Immunoblot verification of AVE0991's attenuation of pyroptosis and RAS dysregulation caused by ACE2 deficiency. (O) Reduction in GSDMD and Caspase-1 fluorescence intensity by AVE0991 treatment. (N) Nucleic acid dyes assessment confirms the protective effect of Ang (1–7) against cell pyroptosis.


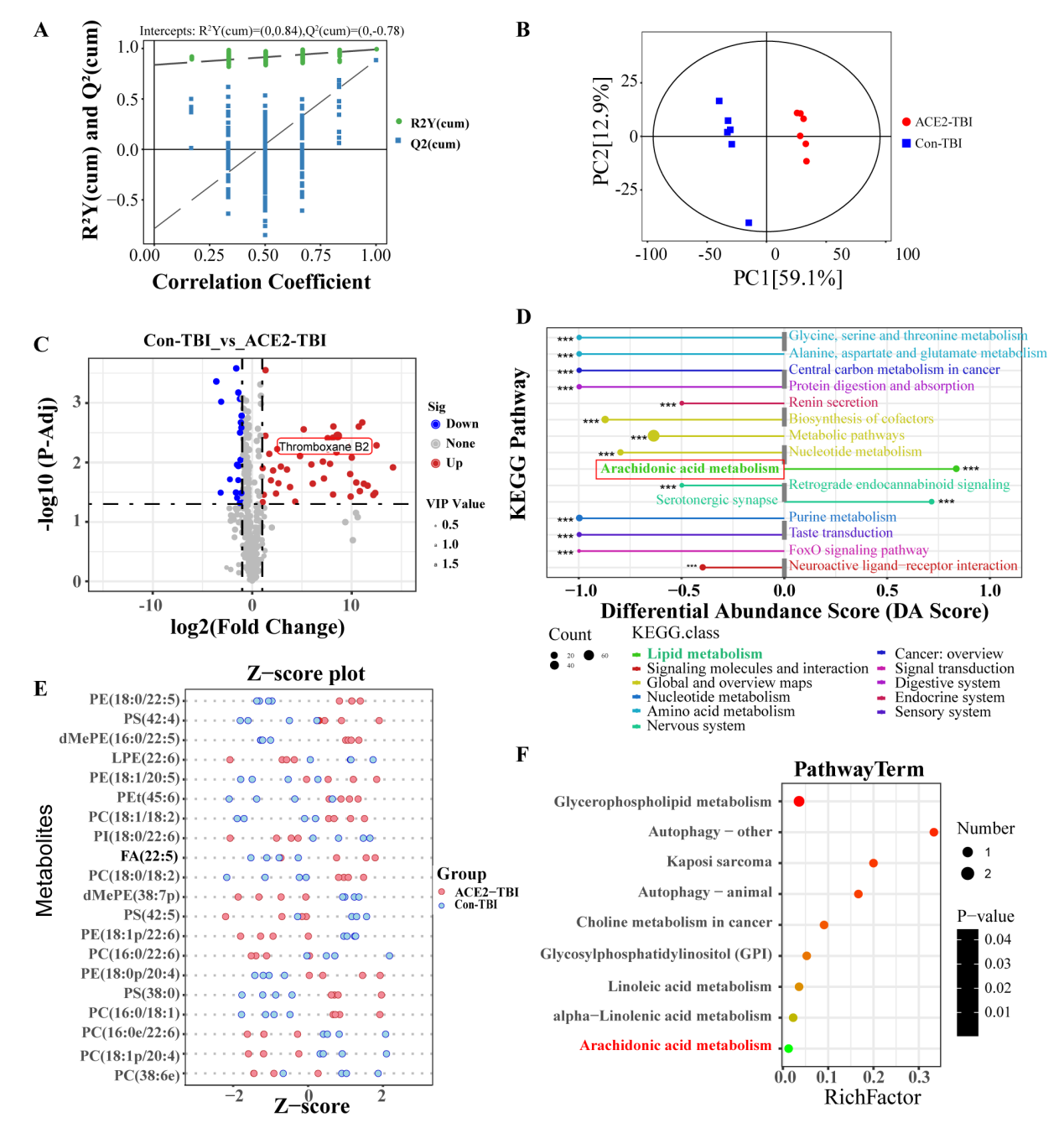


**Fig. S3. Comprehensive Analysis of Metabolic and Proteomic Alterations Associated with ACE2 Deficiency in TBI.**

(A) Dot plot indicating the permutation test results for the OPLS-DA model in untargeted metabolomics. (B) PCA model score scatter plot, where the red dot represents the ACE2-TBI group, and the blue dot represents the Con-TBI group. (C) Volcano plot for differential metabolite screening, with upregulated metabolites in red and downregulated metabolites in blue. (D) Differential abundance (DA) score plot comparing Con-TBI and ACE2-TBI groups, with a score of 1 indicating an uptrend in the expression of annotated differential metabolites within the pathway and the line length representing the absolute DA Score value. (E) Z-score analysis plot of the top 20 significant differential metabolites based on VIP scores. (F) Bubble plot illustrating metabolite pathway enrichment.

**
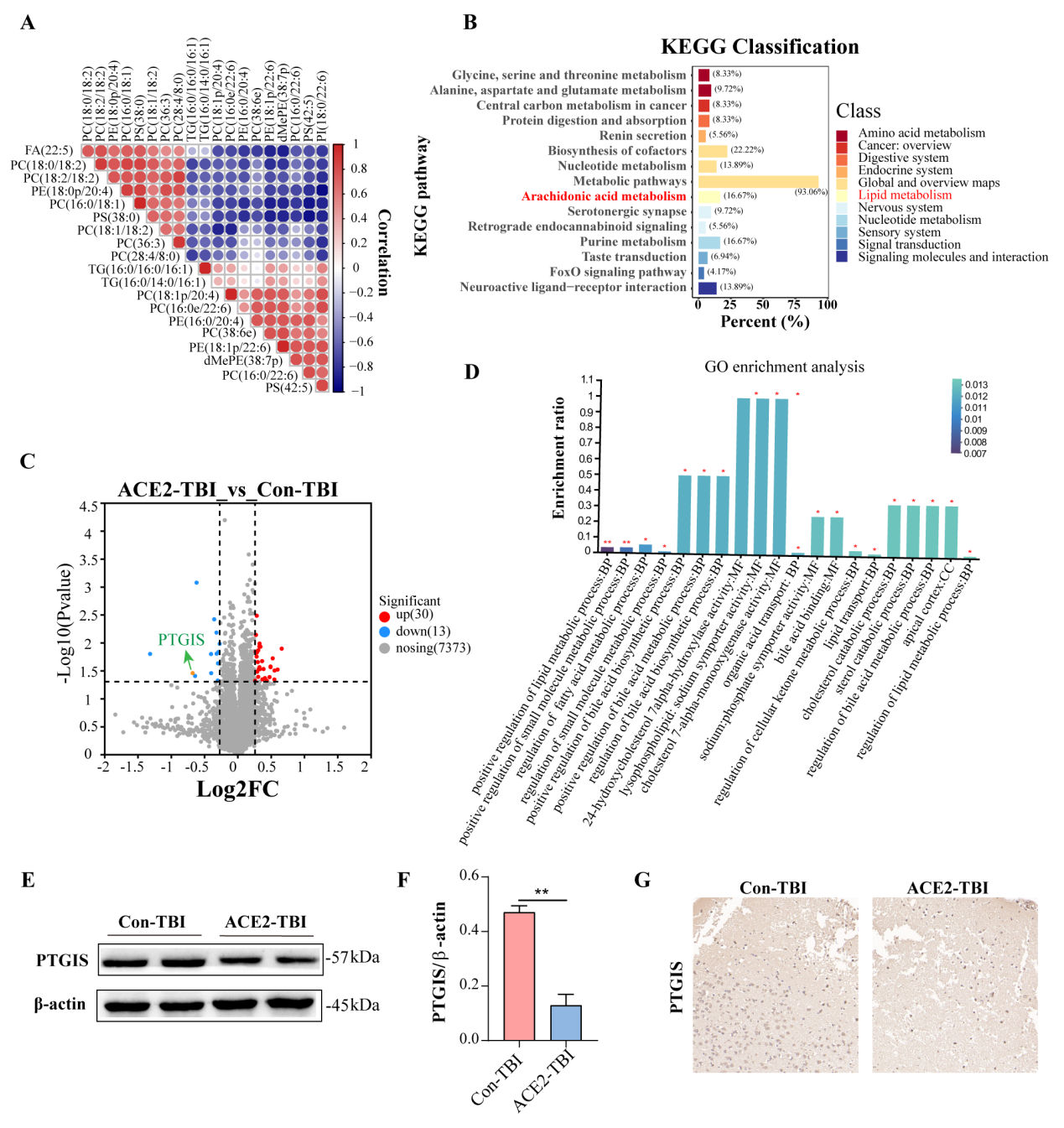
Fig. S4. ACE2 Gene Deletion Induces Lipidomic Dysregulation and Suppresses PTGIS Expression.**

(A) Cluster diagram illustrating differential lipid metabolites. (B) KEGG classification analysis of differential metabolites. (C) Volcano plot depicting upregulated (red) and downregulated (blue) proteins. (D) Schematic representation of GO enrichment analysis for differential proteins. (E-F) PTGIS expression inhibition following ACE2 gene deletion post-TBI. (G) Immunohistochemical localization of PTGIS in the mouse TBI lesion site.


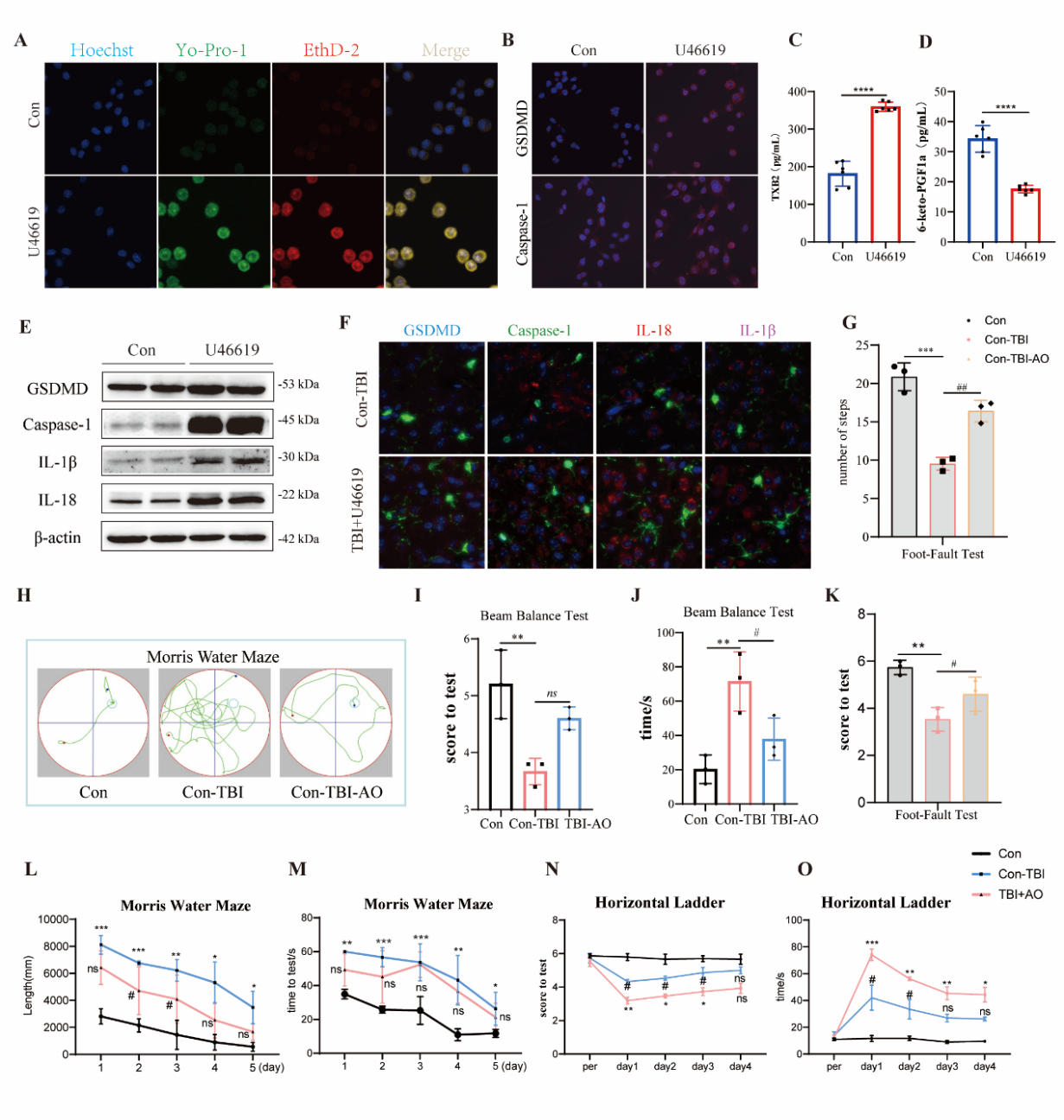


**Fig. S5. TXA2 Exacerbates Cell Pyroptosis, and Ozagrel Alleviates Behavioral Deficits after TBI.**

(A) Nucleic acid dyes analysis of thromboxane A2 agonist U46619’s effects on cell pyroptosis. (B) Representative images indicating U46619’s influence on pyroptosis marker immunofluorescence. (C-D) Analysis of U46619’s impacts on the TXB2 and 6-keto-PGF1a. (E) Western blot validation of the U46619’s impacts on pyroptosis-related protein expressions. (F) U46619 enhances the fluorescence of pyroptotic and inflammatory factors after TBI. (H) Investigation of ozagrel’s effects on learning and cognition post-TBI, with representative traces indicating the mice's paths. (L) Ozagrel reduced the distance traveled in the platform quadrant, though it had minimal effect on the time spent searching for the submerged platform (M). (I–K) Ozagrel improves motor coordination and balance in mice with brain injury, evidenced by shorter completion times in the horizontal ladder and balance beam tests and increased motor scores. (N-O) Ozagrel increases the number of correct stepping behaviors and scores within 60 s in mice. Data are represented as mean ± SEM of three independent experiments.
